# Supplementary material for: MetaRibo-Seq measures translation in microbiomes
Source: Nat Commun. 2020 Jun 29;11:3268. doi: 10.1038/s41467-020-17081-z (PMC7324362; doi:10.1038/s41467-020-17081-z)
Supplement: Supplementary file 10 — Supplementary Data 7 [file 41467_2020_17081_MOESM10_ESM.zip › File2/Confidence_High_Taxonomy/345864_out.krona.html]

Javascript must be enabled to view this page.

members
magnitude
magnitudeUnassigned
count
unassigned
taxon
rank

345864\_out

75

2
superkingdom
75

phylum
1
1239

1
class
186801

order
1
186802

family
1
31979

1485
1
genus


SRS017821\_contig\_number\_25719
species
1
1262773

74
phylum
976

class
74
200643

order
74
171549

815
74
family

74
816
genus
3

SRS013940\_contig\_number\_19370SRS046717\_contig\_number\_contig-100\_1736.71733SRS147139\_contig\_number\_9938

2292923
1

SRS016381\_contig\_number\_2780
species

1

SRS024435\_contig\_number\_21678
species
291644


SRS023346\_contig\_number\_4400SRS043001\_contig\_number\_1326SRS143598\_contig\_number\_21073
species
3
457391

671267
7

SRS022071\_contig\_number\_31602SRS023583\_contig\_number\_13173SRS045713\_contig\_number\_19812SRS048870\_contig\_number\_17426SRS064232\_contig\_number\_4072SRS077127\_contig\_number\_14554SRS148319\_contig\_number\_4783
species

818
species

SRS013215\_contig\_number\_1206SRS015960\_contig\_number\_3356SRS056519\_contig\_number\_952SRS057717\_contig\_number\_2242SRS1041112\_contig\_number\_3673
5


SRS012273\_contig\_number\_40188SRS013476\_contig\_number\_39769SRS015890\_contig\_number\_3047SRS018541\_contig\_number\_12712SRS018984\_contig\_number\_996SRS023715\_contig\_number\_13306SRS024132\_contig\_number\_19682SRS024549\_contig\_number\_449SRS075341\_contig\_number\_12370SRS1055034\_contig\_number\_9454SRS142712\_contig\_number\_5126SRS149784\_contig\_number\_26955SRS149879\_contig\_number\_27599
species
13
1263042

1739517
1

SRS098717\_contig\_number\_2539
species

817
3

SRS1055043\_contig\_number\_25382SRS1055099\_contig\_number\_120SRS140645\_contig\_number\_266
species


SRS012849\_contig\_number\_23339SRS013098\_contig\_number\_32848SRS018133\_contig\_number\_3404SRS020869\_contig\_number\_34494SRS022137\_contig\_number\_18668SRS053649\_contig\_number\_13005SRS058070\_contig\_number\_20645SRS058770\_contig\_number\_15287SRS078419\_contig\_number\_9587SRS1041038\_contig\_number\_16920SRS1041145\_contig\_number\_27188SRS1055076\_contig\_number\_15223SRS893230\_contig\_number\_8091SRS893300\_contig\_number\_8993
species
14
821

1

SRS024663\_contig\_number\_3600
species
2292941

357276

SRS016018\_contig\_number\_898SRS018049\_contig\_number\_276SRS019286\_contig\_number\_5457SRS042628\_contig\_number\_29811SRS045195\_contig\_number\_6205SRS048981\_contig\_number\_2570SRS052078\_contig\_number\_1529SRS054590\_contig\_number\_237SRS064276\_contig\_number\_15918SRS075021\_contig\_number\_contig-100\_2.45623SRS075963\_contig\_number\_contig-100\_197.197SRS098655\_contig\_number\_2113SRS101268\_contig\_number\_251SRS1055067\_contig\_number\_10220SRS148874\_contig\_number\_1214
species
15

2
species

SRS019685\_contig\_number\_21668SRS077024\_contig\_number\_7314
47678

2
species

SRS018575\_contig\_number\_1216SRS104636\_contig\_number\_12010
376806


SRS014613\_contig\_number\_8122SRS048060\_contig\_number\_12413SRS1054691\_contig\_number\_4704
species
3
2292935
